# New alternative approaches to stroke treatment: the blood cell–derived secretome shows promise in individuals with obesity

**DOI:** 10.1007/s11011-024-01491-9

**Published:** 2024-12-06

**Authors:** Klaudia Kotorová, Jana Končeková, Martin Bona, Petra Bonová

**Affiliations:** 1 Biomedical Research Center of the Slovak Academy of Sciences, Institute of Neurobiology, Soltesovej 4–6, 040 01 Košice, Slovak Republic; 2 Department of Medical Physiology, Faculty of Medicine, University of Pavol Jozef Safarik, Košice, 040 01 Slovak Republic

**Keywords:** Stroke, Obesity, Remote ischaemic conditioning, Blood cell-derived secretome, Neuroprotection

## Abstract

Ischaemic tolerance induced by remote ischaemic conditioning (RIC) has been extensively demonstrated in several preclinical models of cerebral ischaemia. However, animals with common stroke-related comorbidities do not benefit from the recent advances of RIC. Therefore, we investigated two alternative approaches for obese animals with stroke: (1) the efficacy of an additional round of the standard RIC protocol, and (2) the paracrine potential of the blood cell–derived secretome derived from RIC-induced healthy young rats. We found that a second round of remote ischaemic postconditioning (RIPostC) stimulus reduced neurodegeneration and exerted antioxidant effects but failed to decrease the infarct volume and alter glutamate homeostasis. However, when obese rats were administered the secretome from healthy, young RIC-stimulated rats, they exhibited improved neurological post-stroke outcomes. Intravenous administration of the tolerant secretome activated several endogenous mechanisms, including a reduction in the infarct volume and neurodegeneration in the penumbra. Furthermore, the blood cell–derived secretome accelerated brain-to-blood glutamate efflux in obese rats, and demonstrated antioxidant properties that may have contributed to the induction of tolerance in obese rats with stroke. These findings indicate that the blood cell–derived secretome has unique abilities and represents a new potential treatment for individuals with obesity and ischaemic stroke.

## Introduction

Ischaemic stroke remains one of the leading causes of long-term disability throughout the world. Considerable effort has been made during the past decades to develop innovative adjuvant therapeutic strategies capable of attenuating brain ischaemia/reperfusion (IR) injury and, consequently, improving stroke outcomes. Over the past decades, a novel, non-invasive strategy has emerged, known as remote ischaemic postconditioning (RIPostC), which induces ischaemic tolerance through several episodes of sublethal IR in distant organs (Zhao et al. 2006). Despite the promising potential of RIPostC, the majority of stroke patients still cannot benefit from the recent advances of remote ischaemic conditioning (RIC) treatment (Hougaard et al. 2014; England et al. 2017; Pico et al. 2020; Landman et al. 2022). This inability to translate RIC from animal models to successful treatment in the clinical setting might be attributed to the large range of comorbidities present in the majority of stroke patients. Indeed, the Global Burden of Disease study and INTERSTOKE, an international case-control study, have shown that modifiable risk factors (such as smoking, poor diet, low physical activity, high BMI, high fasting plasma glucose, high total cholesterol, obesity etc.) contribute to nearly 90% of the burden of stroke, depending on the number of risk factors included (Feigin et al. 2016). Therefore, there is an urgent demand for new treatments that can effectively improve stroke management in patients exposed to those factors, including obesity.

The use of repeated RIPostC may be a simple strategy to minimise the outcomes after ischaemic stroke in obese animals (Ren et al. 2015). Several earlier studies demonstrated the benefits of three cycles of 5 min of ischaemia interspersed with 5 min of reperfusion periods. However, an optimal RIPostC protocol has not yet been identified. In our previous study, we used a conventional postconditioning stimulus (three cycles of 5 min of ischaemia with 5 min of reperfusion), but it provided limited protection in obese rats (Kotorová et al. 2024). Accordingly, our first aim was to investigate the effect of a second round of standard RIPostC in Zucker diabetic fatty (ZDF) rats subjected to ischaemic stroke. We hypothesised that as the dose of RIPostC increases, the magnitude of the benefits compared with the standard dose increases. Our second aim is based on our previous findings, where the secretome prepared from the blood cells underwent the remote ischemic conditioning has effectively built the tolerant phenotype as a pre- or post-stroke treatment when the young healthy individuals were used in experiments (Bonova et al. 2020). The main focus of the presented results was to test the injection of such prepared blood cell–derived secretome from healthy rats into ZDF rats and to determine the severity of neurological deficits and neurological outcomes after stroke. Thus, we explored the underlying mechanisms responsible for the neuroprotective effects of the secretome to better understand these mechanisms and contribute to the development of more targeted therapeutic approaches for ischaemic stroke in the context of obesity.

## Materials and methods

### Ethics statement and animal use

All animal experiments were conducted in accordance with the approved animal care guidelines outlined in the European Communities Directive 2010/63/EU. These experiments were conducted with the authorisation of the State Veterinary and Food Administration in Bratislava (2978-5/2021 − 220 and 4518/19–221/3) under the supervision of the Ethical Council of Neurobiology BMC SAS. Wistar albino normal-weight rats (250–300 g, obtained from Velaz Ltd., Czech Republic) and ZDF rats (350–450 g, obtained from Dobrá Voda, Slovakia) were housed in standard conditions (20 ± 2 ºC, 55% ± 5% relative humidity and a 12-h light/dark cycle, and were provided food and water *ad libitum*. The experiments were conducted with 12-week-old male rats to minimise the influence of non-modifiable risk factors for stroke, such as older age and sex. All efforts were taken to minimise suffering.

### Experimental design

Each experimental group contained 10 animals, although the minimum number of animals per group was set at 6 due to spontaneous death caused by cerebral ischemia as calculated previously (Bonova et al. 2016, 2020). The control group was set to 6 rats.

The study was divided into two sub-studies. For the first sub-study, the effect of a second round of RIPostC in ZDF rats was evaluated. Prior to surgery, rats were randomly assigned to one of the following four groups: control, ischaemia, tolerant and double tolerant. Rats from the ischaemia, tolerant and double-tolerant groups were subjected to 90 min of middle cerebral artery occlusion (MCAO) using an intraluminal filament technique developed by Longa et al. (Longa et al. 1989). The tolerant rats were subjected to one round of RIPostC, consisting of three cycles of 5 min of ischaemia– induced by placing an elastic rubber band tourniquet around the hind limb – followed by 5 min of reperfusion. This procedure was performed within 1 h after MCAO. A second round of the same RIPostC protocol was administered within 1 h after the previous IR cycle to the double-tolerant rats.

For the second sub-study, a tolerant phenotype in ZDF rats was induced by administering the secretome derived from blood cells of lean, healthy Wistar rats subjected to one round of RIPostC (Tolerant secretome group). The blood cell–derived secretome of the tolerant animals was prepared as described below and then intravenously injected into the ZDF rats within 30 min after transient ischaemia (90 min of MCAO). The effect of tolerant secretome was compared with non-tolerant secretome derived from blood cells of lean, healthy Wistar rats non-subjected to RIPostC (Non-tolerant group). The control rats represented intact ZDF rats (Control group). The rats were euthanised 24 h after the surgery under deep anaesthesia induced by 4% isoflurane.

###  Middle cErebral Artery Occlusion (MCAO) model

MCAO induction followed the procedure described by Longa et al. (Longa et al. 1989). The rats were anaesthetised using 4% isoflurane. A commercially available 6 − 0 monofilament nylon suture, which had a silicone rubber-coated tip, was inserted through an incision made in the external carotid artery into internal carotid artery to occlude the origin of the right middle cerebral artery (MCA) for 90 min. To effectively block the MCA, the filament was inserted approximately 19–20 mm from the carotid bifurcation. Cerebral blood flow was monitored using a laser-Doppler flow meter (Periflux System 5000, Perimed AB, Sweden). A 407 probe, along with an appropriate holder, was placed on the skull over the MCA (5 mm lateral and 1 mm posterior to bregma). Only rats whose blood flow dropped below 80% following filament insertion were included in the study.

### Remote ischemic postconditioning

RIPostC was performed within 60 min after MCAO. The rats were subjected to three cycles of 5 min of ischaemia followed by 5 min of reperfusion of the femoral artery by placing an elastic rubber band tourniquet around the hind limb in a tight position to occlude the arterial blood supply.

### Preparation of the blood cell–derived secretome

The blood of the RIC-stimulated (tolerant) and unstimulated (non-tolerant) Wistar rats was collected by cardiac puncture and processed as it was described previously (Bonova et al. 2016, 2020). After centrifugation (4500 *g*, 15 min, 4 °C), the blood plasma was discarded. To retain the original blood volume, the pellet, which represented a mix of all blood cell types, was washed and resuspended in artificial plasma (hydroxyethyl starch [HES] 130/0.4 in an isotonic electrolyte solution, 6% Volulyte, Fresenius Kabi Deutschland GmbH). Subsequently, the samples were incubated in a CO_2_ incubator (37 °C for 150 min) followed by centrifugation (4500 *g*, 15 min, 4 °C). The supernatant was removed and centrifuged again (15,000 *g*, 15 min, 4 °C) to remove any remaining cells and cellular debris. The final supernatant – the blood cell–derived secretome with preserved exosomes and microparticles – was injected intravenously into ZDF rats that had undergone 90 min of MCAO according to Longa et al. (Longa et al. 1989), as mentioned previously.

### Brain sample preparation

After decapitating the animals, as described previously, their brains were rapidly removed and cut into 2-mm coronal slices, which were used for 2,3,5-triphenyltetrazolium chloride (TTC; Sigma), FluoroJade B (FJB; Histo-Chem Inc., USA) and haematoxylin & eosin (H&E) staining. For the tissue analysis of glutamate, slices were dissected from the ischaemic core and penumbra of the ipsilateral and contralateral hemispheres, as described previously (Ashwal et al. 1998), with minor modifications. Subsequently, the tissue was weighed, homogenised in homogenisation buffer (20 mM Tris-HCl pH 7.5 containing 1 mM DTT, 50 mM magnesium acetate, 140 mM KCl, 1 mM EDTA, 2 mM EGTA with the addition of Protease Inhibitor Cocktail Tablets, Roche, Germany) and centrifuged at 12,000 rpm for 15 min at 4 ºC. The total protein concentration was determined using the method described by Bradford (Bradford 1976), using a standard curve established with bovine serum albumin (BSA). The core and penumbral post-mitochondrial supernatants were precipitated with 1 M ice-cold perchloric acid (PCA; 1:20; 10 min, 4 ºC), and centrifuged (12,000 rpm, 10 min, 4 ºC). The supernatant was collected and stored at −80 ºC for later analysis.

### Blood sample processing

Whole blood samples were obtained by cardiac puncture and collection in heparin-coated tubes 24 h after the ischaemia. Blood samples were centrifuged at 12,000 rpm for 10 min at 4 ºC to separate plasma and blood cells. Catalase (CAT) activity was measured in the plasma. Superoxide dismutase (SOD) activity was measured in the blood cell lysate. The blood cell pellet was suspended in distilled water equivalent to the volume of plasma collected. After rapid freezing (10 min, −20 ºC) and defrosting with three cycles at 37 ºC, the samples were centrifuged at 12,000 rpm for 10 min at 4 ºC. The supernatant, representing the blood cell lysate, and plasma samples were stored at −80 ºC until further analysis.

Lymphocytes assigned for the comet assay were isolated from 100 µl of whole blood and diluted in phosphate-buffered saline (PBS) at a 1:4 ratio. Isolation was performed via density centrifugation (2000 rpm, 5 min, 4 ºC) using a Ficcol–Paque™ plus gradient. The layer containing lymphocytes was collected, rewashed in PBS (1:4), and directly used for assessing DNA via Single-Cell Gel Electrophoresis.

Glutamate was measured in the whole blood. Samples were deproteinised by adding ice-cold 1 M PCA at a 1:1 ratio, precipitated for 10 min on the ice and centrifuged at 12,000 rpm for 10 min at 4 ºC. The supernatant was collected and stored at −80 ºC for subsequent analysis.

### Evaluation of the infarct volume and neurological deficits

After dividing the brain into 2-mm-thick coronal sections, these sections were promptly placed in a solution consisting of 2% TTC in PBS at 37 ºC for 30 min for vital staining. Subsequently, they were immersed in a 10% formaldehyde solution for fixation for 24 h. Each section was scanned using a high-resolution scanner (Epson Perfection 4490 Photo, resolution 600 dpi), and digital images of five slices from each brain were analysed using Image J software by blinded investigator (version 1.8.0, National Institute of Health, Bethesda, MD, USA). The extent of brain damage was quantified by determining the ratio of the infract area (white area on the right side) to the area of the undamaged, contralateral hemisphere. The size of infarct was calculated using the following equation: infarct rate (mm^3^) = (non-ischaemic volume/ischaemic volume) × infarct volume (Callaway et al. 2000).

The animals were neurologically assessed 1 and 24 h after reperfusion. Evaluation and scoring was blind, without experimenter knowledge of rat’s membership in group. A four-point scale described by Bederson et al. (Bederson et al. 1986) was used for neurological assessment: (1) forelimb flexion and torso turning to the contralateral side when lifted by the tail; (2) the same behaviour as grade 1 and decreased resistance to a lateral push; (3) the same behaviour as grade 2 with unilateral circling; and (4) no spontaneous walking and a depressed level of consciousness. Each category was scored from 0 to 3 (the greater the score, the more severe the impairment). Rats with neurological deficits lower than 2 were excluded from the study.

### Histology

After TTC labelling, the fixed brains were dehydrated in a 30% sucrose in PBS. They were sliced into 25-µm-thick coronal sections using a Leica CM1850 cryostat and mounted directly on gelatine-coated slides. The sections were allowed to air dry and were subsequently rehydrated in decreasing concentrations of ethanol (from 100 to 50%). Following rehydration, the sections were stained using the H&E Staining Kit (Abcam, Cambridge, UK). After staining, the tissue sections were rinsed with distilled water, dehydrated through a series of ethanol solutions (from 70 to 100%), cleared in xylene and mounted with mounting solution (DPX Mountant; Fluka Chemie AG, Switzerland). Once the mounting solution had solidified, images were captured using the Aperio AT2 digital scanner (Leica Biosystems, Wetzlar, Germany). Ischaemic damage was assessed and quantified as the infarct volume by measuring the areas of cell death, as described previously (Osborne et al. 1987), and by counting pyknotic cells in the striatum and cortex within the penumbra per mm^2^ using the ImageJ software.

### Fluoro-Jade B staining

Twenty-five-micrometre-thick coronal sections were prepared following the same procedure as for H&E staining. The sections mounted on gelatine-coated slides, air-dried and then heated at 50 ºC for at least 15 min before staining. The slides were immersed in absolute alcohol, 70% ethanol and for 3 min in distilled water. The sections were then placed in a 0.06% potassium permanganate solution for 15 min and subsequently rinsed in distilled water for 2 min. Afterwards, the sections were transferred into a 0.0001% solution of FJB dissolved in 0.1% acetic acid for 30 min. The slides were washed three times with distilled water, left to air dry overnight at room temperature, cleared in xylene and then cover-slipped with DPX Mountant (Fluka Chemia AG). The slides were examined using an Olympus BX51 microscope equipped with an Olympus DP50 camera. FJB-positive neurons within the ischaemic penumbra were counted in 10 randomly selected 1-mm^2^ areas and expressed per mm^2^ of tissue using the ImageJ software.

### Glutamate concentration

The glutamate concentration in blood and brain tissue was measured by using a modified enzymatic fluorimetric method described by Graham and Aprison (Graham and Aprison 1966; Kravcukova et al. 2009). The method is based on the fluorimetric detection of NADH produced by the glutamate and NAD + reaction catalysed by glutamate dehydrogenase. The glutamate concentration in the reaction is directly proportional to the NADH concentration. Briefly, 10 µl of supernatant, 190 µl of reaction buffer (0.25 M hydrazine hydrate/0.3 M glycine buffer, pH 8.6) containing 200 nM NAD + and 15 U of glutamate dehydrogenase were pipetted into a black 96-well plate. The fluorescence intensity of the final product (NADH) was measured using a Synergy™ 2 Multi-Mode Microplate Reader (BioTek) at 460 nm and an excitation wavelength of 360 nm 30 min after incubation at room temperature. The glutamate blood concentration is expressed as µmol/l blood, and the glutamate brain concentration was normalised according to the protein content and is expressed as µmol/mg protein.

### Comet assay

The alkaline comet assay was performed based on the method described by Singh et al. (Singh et al. 1988), with minor modifications. First, microscope slides were covered with 1% normal-melting-point agarose in PBS (pH 7.4) and allowed to dry at room temperature. A suspension of lymphocytes was mixed with 1% low-melting-point agarose in PBS (pH 7.4). This mixture was spread onto slides precoated with agarose, covered with coverslips and allowed to dry in a refrigerator for 2 min. The coverslips were then removed, and the slides were immersed in a lysis solution (containing 2.5 mol/l NaCl, 100 mmol/l Na_2_EDTA, 10 mmol/l Tris, 1% Triton X-100 and 10% DMSO, pH 10) for 1 h at 4 ºC. Subsequently, the slides were transferred to an alkaline electrophoresis buffer (comprising 5 mol/l NaOH and 200 mmol/l Na_2_EDTA, pH 13) for 20 min at 4 ºC, and electrophoresis was performed at 25 V for 25 min at 4 ºC. Then, the slides were neutralised in a neutralisation buffer solution (containing 400 mmol/l Tris, pH 7.5) for 15 min at 4 ºC. After air-drying, DNA was stained with SYBR Green, and 100 cells on each slide were examined under a fluorescence microscope (Olympus BX51, excitation filter at 485 nm, emission filter at 520 nm) equipped with a camera (Olympus DP50). The images were analysed using the Comet Score™ v1.5 image analysis system (TriTek Corp., USA). DNA damage was assessed based on the parameter ‘% DNA in tail’, calculated as the difference between 100% of cell fluorescence intensity and the intensity within the head region, and represented as a percentage.

### Determination of antioxidant enzyme activities

#### Superoxide dismutase

Superoxide dismutase (SOD) activity was measured according to the method described by Sun et al. (Sun et al. 1988). The standard assay substrate mixture contained the following (in 200 µl): 1 M xanthine (Sigma), 0.1 M EDTA, 5.6 × 10^−2^ M *p*-nitrotetrazolium (NBT, blue grade III, Sigma-Aldrich, Steiheim, Germany) and 1 M BSA (Fluka) in 0.1 M sodium phosphate (pH 7.8). This assay employs xanthine-xanthine oxidase as a source of superoxide to prevent the reduction of NBT by superoxide. It quantitatively measures the absorbance of NBT, which is converted into a blue formazan compound by superoxide, at 560 nm and room temperature. SOD in the sample breaks down superoxide, slowing the production of blue formazan. In the context of blood cells, one unit of SOD activity is defined as the amount that reduces the absorbance change by 50%.

#### Catalase

Catalase (CAT) activity in erythrocytes was quantified using spectrophotometry, following the method described by Góth (Góth 1991). This method is based on the creation of a stable complex between hydrogen peroxide and ammonium molybdate. In brief, 20 µl of the sample was mixed with 100 µl of the substrate (65 µmol/ml of hydrogen peroxide in a 60 nmol/l sodium potassium phosphate buffer at pH 7.4) for 60 s at 37 ºC. A stop solution, comprising ammonium molybdate (32.4 mmol/l; 100 µl), was used, and the yellow complex formed by molybdate and hydrogen peroxide was measured at 405 nm. One unit of CAT activity is defined as the quantity of enzyme required to break down 1 µmol of hydrogen peroxide within 1 min under these specific conditions.

#### Glutathione peroxidase

Glutathion peroxidase (GSH) activity was measured in the whole blood sample using the colorimetric method described by Alisik et al. (Alisik et al. 2019), with minor modifications. A sample of precipitated blood was incubated with 10 mM Ellman’s reagent in 500 mM Tris buffer (pH 8.2) with 10 mM EDTA for 10 min at room temperature. The absorbance was measured at 415 nm after the incubation. Glutathione (0–15 nM) was used as a calibrator for the assay.

### Statistical analysis

Data were analysed and plotted using GraphPad Prism (Graph-Pad Software, San Diego, CA, USA). Statistical analysis was performed using one- and two-way analysis of variance, followed by the Dunnett post hoc test. The criterion for statistical significance was *p* < 0.05.

## Results

### Sub-study 1

#### The effect of a second round of RIPostC on the infarct volume and morphology of MCAO-induced brain of obese rats

**Fig. 1:**
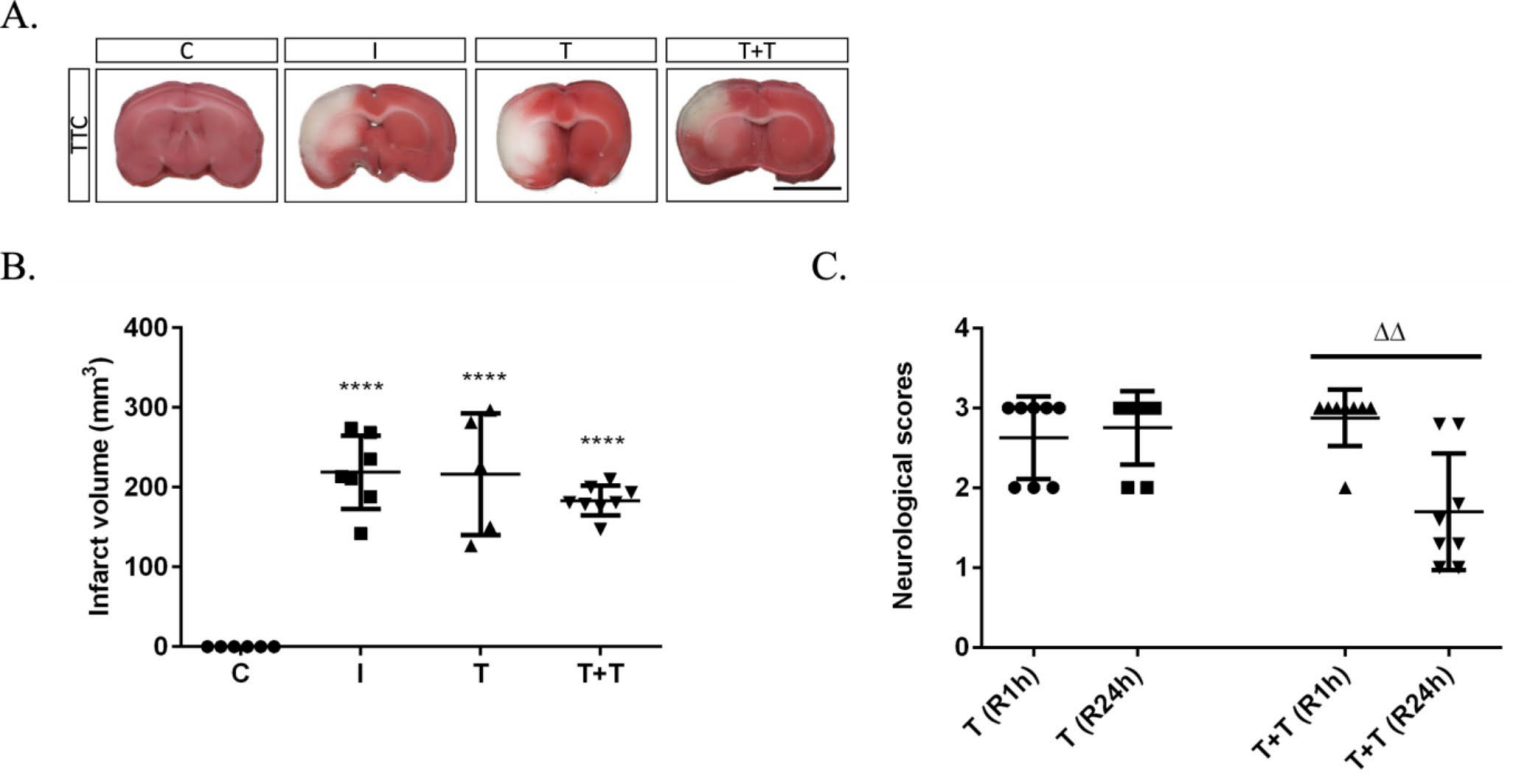
(**A**) Representative TTC-stained 2-mm brain slices from the control (C), ischaemia (I), one round of RIPostC (tolerant; T) and two rounds of RIPostC (double tolerant; T + T) treated obese rats. The scale bar is 10 mm. (**B**) This histogram shows quantification of the infarct volume. (**C**) The histogram shows neurological function measurement following one round or two round of RIPostC after 1 h (R1h) and 24 h (R24h) of reperfusion. The data are presented as the mean ± SD. ****p < 0.0001 versus the control group; ^ΔΔ^*p* < 0.01 (‘Δ’ represents a statistically significant R1h versus R24h group difference)

Compared with the ischaemia group (218.80 ± 17.43 mm^3^; *n* = 7), the brain infarct volume of ZDF rats that were exposed to two rounds of conventional RIPostC (*n* = 8) was reduced to 183.30 ± 6.65 mm^3^. Although the mean infarct volume after a second round of RIPostC was smaller than what we observed after a single round of RIPostC episodes (201.10 ± 31.69; *n* = 6), a total of six cycles did not lead to a significant reduction in the infarct volume compared with the ischaemia group (Fig. 1A, B). Nonetheless, neurological assessments indicated a significant improvement following the second round of RIPostC (*p* < 0.0031) (Fig. 1C).

**Fig. 2:**
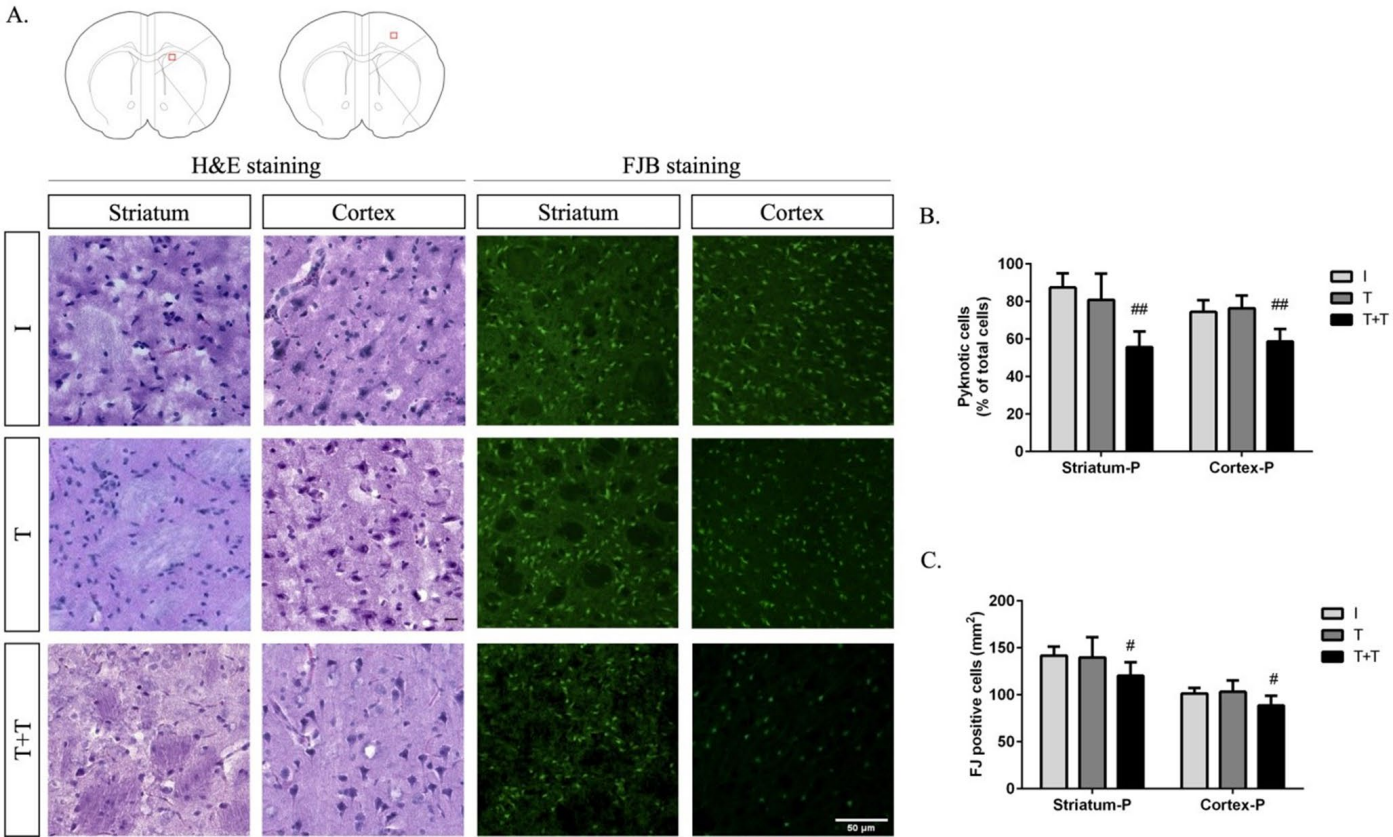
(**A**) The photomicrographs show H&E and FJB staining indicating neurodegeneration in the striatum and cortex 24 h after ischaemia (I), one round of RIPostC (tolerant; T) and two rounds of RIPostC (double tolerant; T + T). The scale bar is 50 μm. The histograms show (**B**) the percentage of pyknotic cells and (**C**) the average number of FJB-positive cells per mm^2^ in the striatum and cerebral cortex within the penumbra. The data are presented as the mean ± SD. ^#^*p* < 0.05 and ^##^*p* < 0.01 versus the ischaemia group

In addition to the infarct volume analysis, we conducted H&E and FJB staining (Fig. 2A). We identified ischaemic changes indicative of cell death, characterised by nuclear pyknotic changes, in 87.43% ± 2.88% of the striatum and 74.43% ± 2.34% of the cortex within the penumbra of rats subjected to MCAO. One round of RIPostC did not significantly affect the percentage of pyknotic cells in either region (80.83% ± 5.75% and 76.33% ± 2.81%, respectively). However, the addition of a second round of RIPostC led to a significant decrease in the percentage of pyknotic cells in both the striatum and cortex (55.70% ± 4.15% and 58.59% ± 3.41%, respectively) (Fig. 2B). Furthermore, brain sections from rats treated with two rounds of RIPostC showed significantly fewer FJB-positive cells in the striatum (*p* < 0.0102) and cortex (*p* < 0.0247) compared with the ischaemia group. There were no significant differences in the number of FJB-positive cells after one round of RIPostC (Fig. 2C).

#### A second round of RIPostC did not affect glutamate homeostasis in obese rats after stroke

**Fig. 3:**
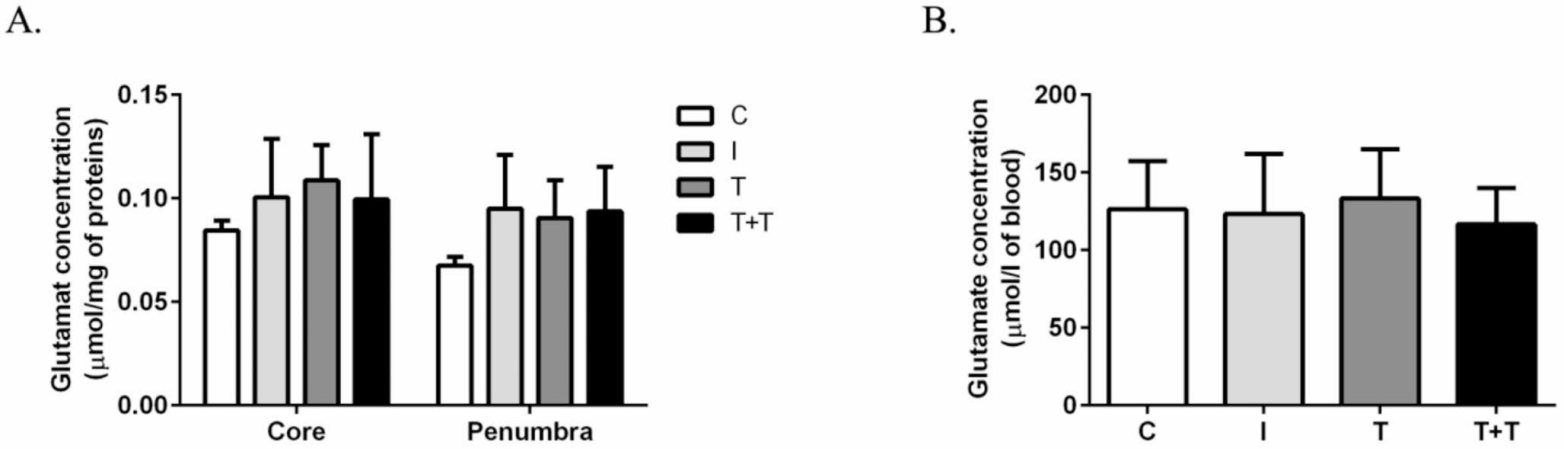
(**A**) The histograms show the glutamate levels in the brain tissue of the ipsilateral core and penumbra and (**B**) the peripheral blood after 24 h of post-ischaemic reperfusion in the control (C), ischaemic (I), tolerant (T) and double tolerant (T + T) groups

As we observed previously, MCAO for 90 min did not have a significant impact on tissue and blood glutamate levels in obese rats. Compared with the ischaemia group (0.100 ± 0.01 µmol/mg of protein in core; 0.095 ± 0.01 µmol/mg of protein in penumbra; 123.21 ± 10.39 µmol/l of blood; *n* = 7), administering one round of RIPostC (*n* = 8) or two rounds of RIPostC (*n* = 8) did not significantly reduce glutamate levels in either the core (0.109 ± 0.01 and 0.100 ± 0.01 µmol/mg of protein, respectively) or the penumbra (0.091 ± 0.01 and 0.094 ± 0.01 µmol/mg of protein, respectively) (Fig. 3A), nor did it result in a significant alteration in the glutamate blood level (133.25 ± 8.83 and 116.60 ± 7.77 µmol/l of blood, respectively) (Fig. 3B).

#### The antioxidant effects of a second round of RIPostC on obese MCAO-induced rats

**Fig. 4:**
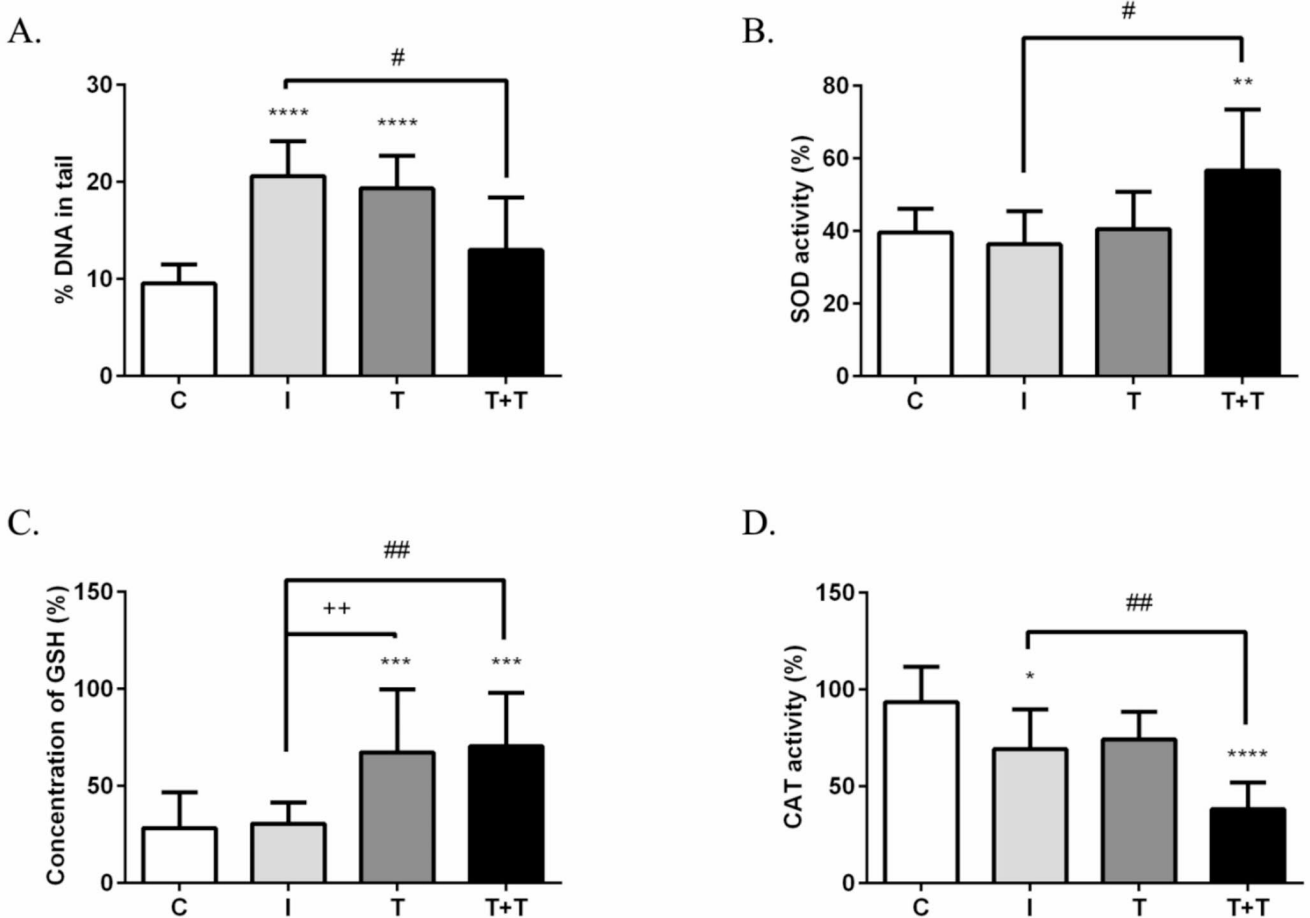
The histograms show quantification of (**A**) single-strand DNA breaks in lymphocytes and (**B**) SOD, (**C**) GSH and (**D**) CAT activities in the blood of control (C), ischaemia (I), one round of RIPostC (tolerant; T) and two rounds of RIPostC (double tolerant; T + T) obese animals. The data are presented as the mean ± SD. *p < 0.05, **p < 0.01, ***p < 0.001 and ****p < 0.0001 versus the control group; ^#^*p* < 0.05 and ^##^*p* < 0.01 versus the ischaemia group

We conducted the comet assay to examine the effect of a second round of RIPostC on single-strand DNA breaks in circulating lymphocytes of obese animals, assessing the percentage of DNA in the tail of comets. At the same time, we measured the levels of antioxidant enzymes, specifically SOD, CAT and GSH. Lymphocytes from rats treated with two rounds of RIPostC (*n* = 7) exhibited a significant reduction in DNA damage (13.00% ± 2.20% DNA in tail) compared with the ischaemia group (20.62% ± 1.46%; *n* = 7) (Fig. 4A). Furthermore, this treatment increased the SOD (Fig. 4B) and GSH (Fig. 4C) activities by approximately 55.00% and 131.08%, respectively, compared with the ischaemia group, and by about 43.00% and 149.00%, respectively, compared with the control group. In contrast, two round of RIPostC further reduced CAT activity by 44.00% compared with the ischaemia group and by 59.00% compared with the control group (Fig. 4D). Treatment with a single round of RIPostC only resulted only in a significant increase in GSH activity, by 120.31% compared with the ischaemia group and by 137.67% compared with the control group (Fig. 4C).

### Sub-study 2

#### The tolerant secretome improved post-stroke outcomes in obese rats

**Fig. 5:**
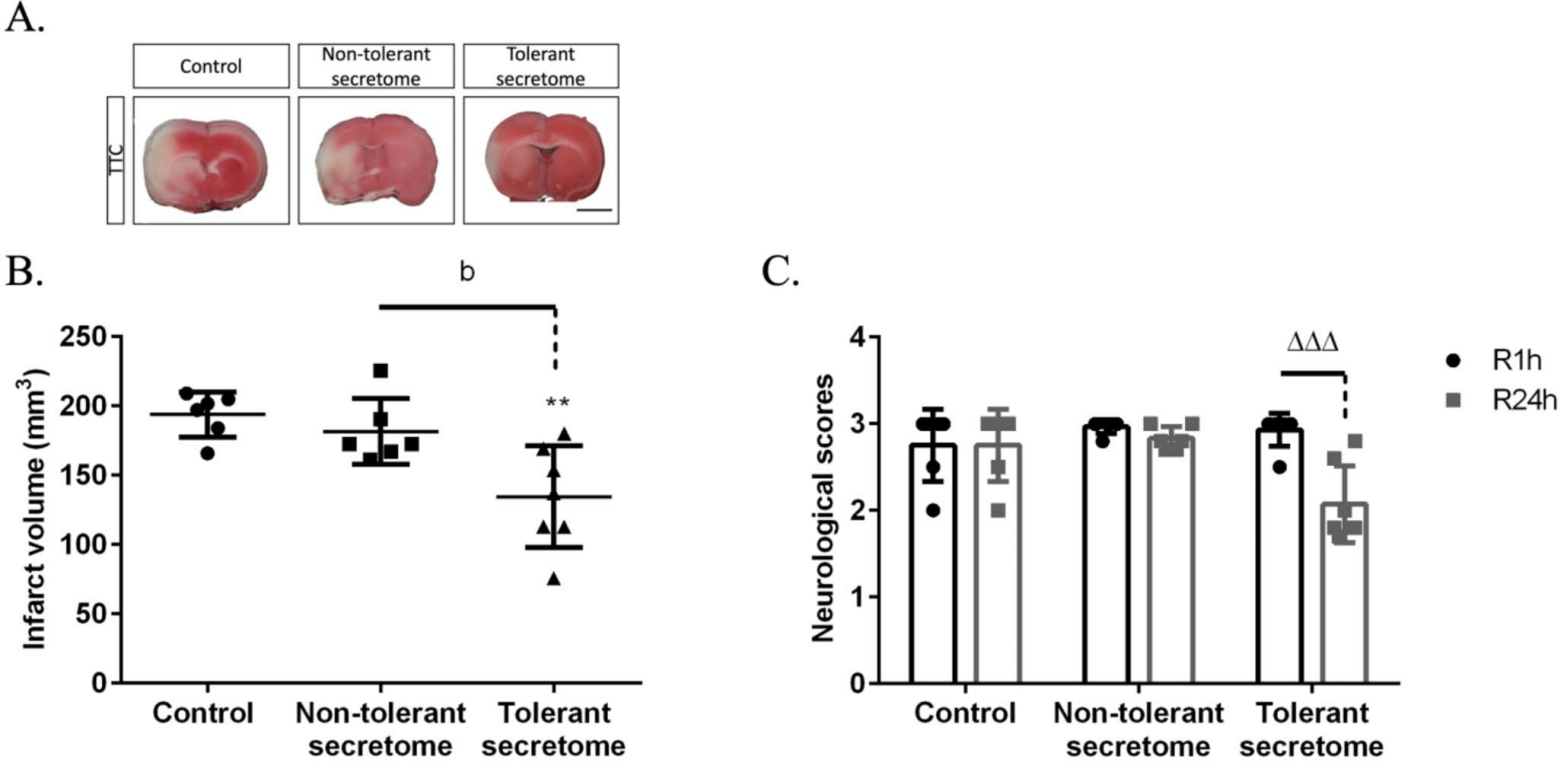
(**A**) Representative TTC-stained images of rat coronal brain sections. The scale bar is 10 mm. The histograms show quantification of (**B**) the infarct volume and (**C**) the neurological scores 1 h (R1h) and 24 h (R24h) following MCAO. The data are presented as the mean ± SD. **p < 0.01 versus the control group; ^b^*p* < 0.05 versus the non-tolerant secretome group; ^ΔΔΔ^*p* < 0.001 (‘Δ’ represents a statistically significant R1h versus R24h group difference)

As a control, we injected MCAO-induced ZDF rats with artificial plasma (*n* = 6). In this group, the infarct size was 193.80 ± 6.59 mm^3^. Administering the non-tolerant secretome (*n* = 6) decreased the infarct volume to 181.60 ± 9.65 mm^3^. However, application of the blood cell–derived tolerant secretome (*n* = 7) significantly decreased the infarct volume to 134.60 ± 13.83 mm^3^, representing a 30.55% reduction compared with the control group and a 25.88% reduction compared with the non-tolerant secretome group (Fig. 5A, B). Moreover, neurological deficits improved significantly after 24 h of reperfusion in the tolerant secretome–treated group (Fig. 5C).

**Fig. 6:**
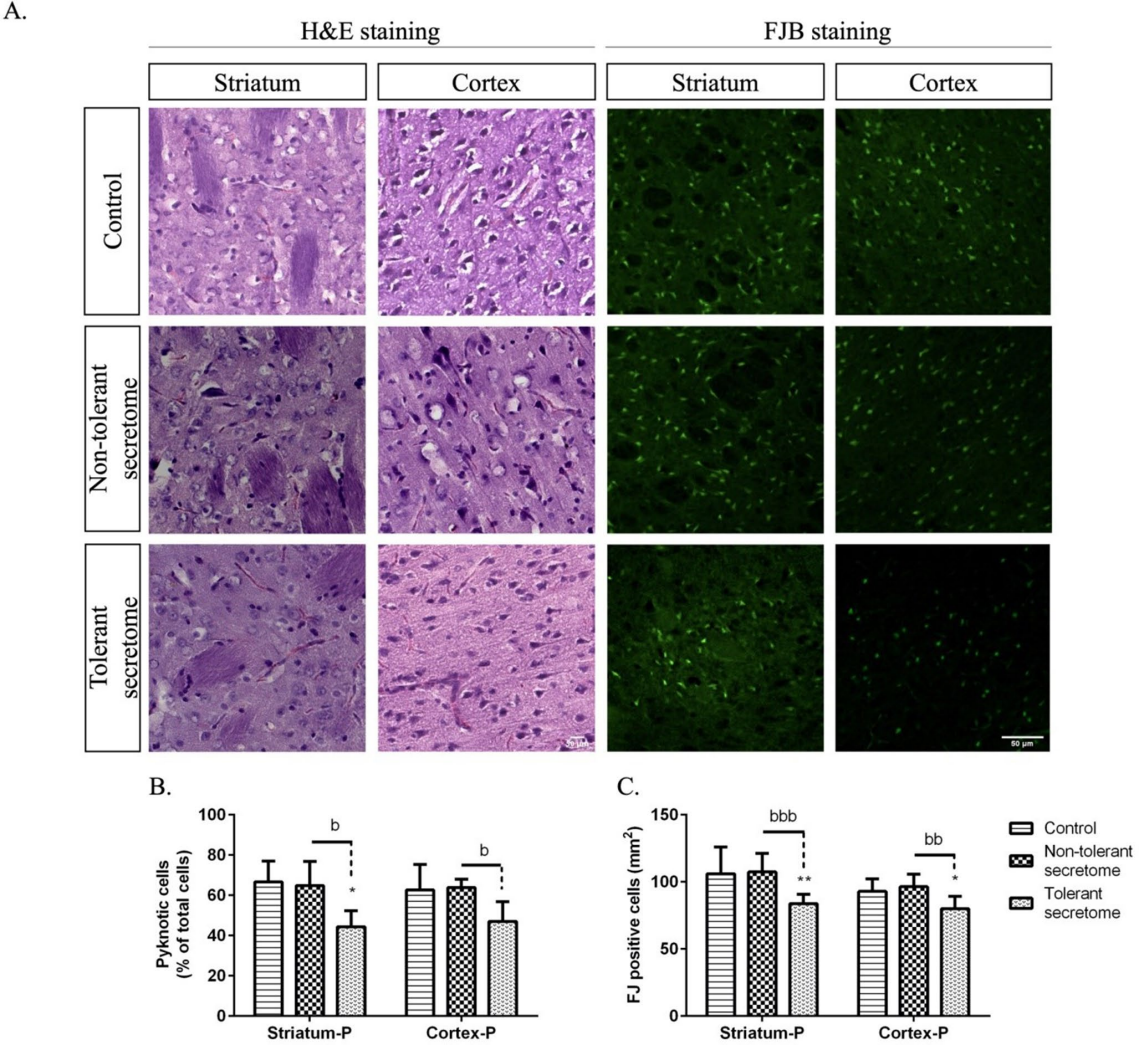
(**A**) The photomicrographs show H&E and FJB staining indicating neurodegeneration in the striatum and cortex within the penumbra. The scale bar is 50 μm. The histograms show quantification of (**B**) the percentage of pyknotic cells and (**C**) the average number of FJB-positive cells per mm^2^ in the striatum and cerebral cortex within the penumbra. The data are presented as the mean ± SD. *p < 0.05 and ** < 0.01 versus the control group; ^b^*p* < 0.05, ^bb^*p* < 0.01 and ^bbb^*p* < 0.001 versus the non-tolerant secretome group

The morphological examination using H&E staining (Fig. 6A) revealed that the mean percentage of pyknotic cells in the control rats did not differ significantly from those exposed to non-tolerant secretome in the striatum (66.73% ± 5.12% and 64.83% ± 6.01% pyknotic cells, respectively) and cortex (62.74% ± 6.31% and 63.78% ± 2.08% pyknotic cells, respectively) within the penumbra. Notably, the tolerant secretome induced a significant reduction to 44.39% ± 3.96% pyknotic cells in the striatum and to 47.08% ± 4.88% pyknotic cells in the cortex within the penumbra (Fig. 6B). We also analysed the brain tissue slices with FJB staining to detect neurodegeneration (Fig. 6A). Following post-ischaemic treatment with the tolerant secretome, there was a significant reduction in FJB-positive cells in the striatum (*p* < 0.0088) and cortex (*p* < 0.0128) within the penumbra compared with the control group. Additionally, there was a significant reduction compared with the group treated with the non-tolerant secretome (*p* < 0.0006 for the striatum, and *p* < 0.0024 for the cortex) (Fig. 6C).

#### The blood cell–derived secretome protects brain from glutamate-induced excitotoxicity

**Fig. 7:**
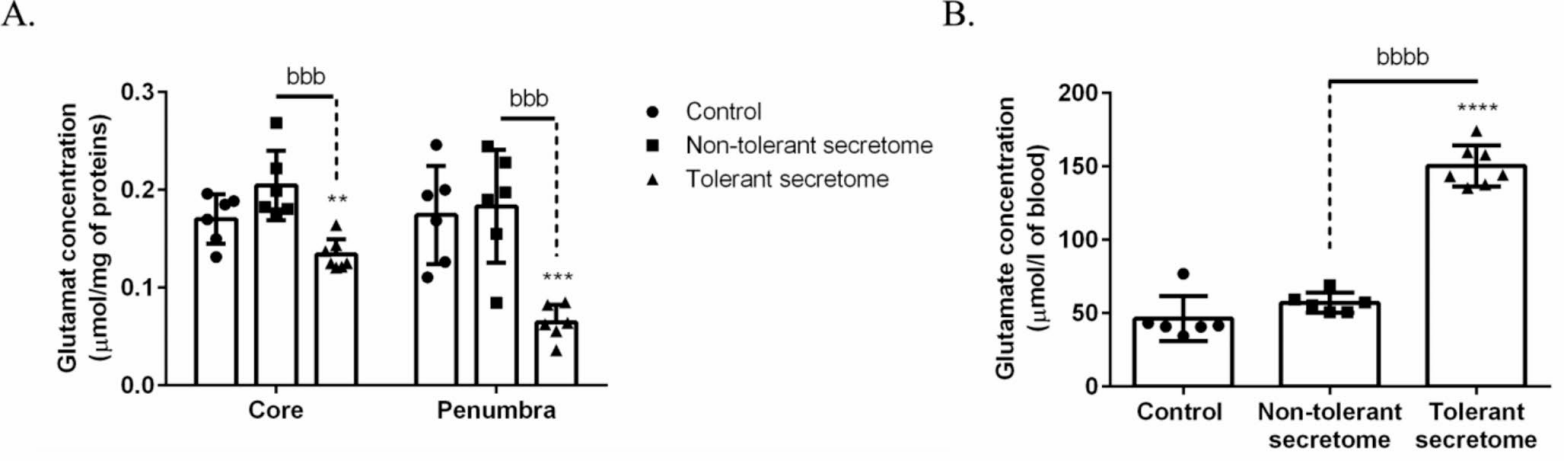
The histograms show quantification of the glutamate levels in (**A**) the ipsilateral core and penumbra and (**B**) peripheral blood after 24 h of post-ischaemic reperfusion. The data are presented as the mean ± standard deviation. **p < 0.01, ***p < 0.001 and ****p < 0.0001 versus the control group; ^bbb^*p* < 0.001 and ^bbbb^*p* < 0.0001 versus the non-tolerant secretome group

Compared with the control group (*n* = 6), the glutamate levels in the core (0.170 ± 0.01 µmol/mg of protein) and penumbra (0.174 ± 0.02 µmol/mg of protein) did not change significantly following treatment with the non-tolerant secretome (0.205 ± 0.01 µmol/mg of protein and 0.183 ± 0.02 µmol/mg of protein, respectively; *n* = 6). In contrast, administering the tolerant secretome (*n* = 7) significantly reduced glutamate by 21.18% in the core (0.134 ± 0.01 µmol/mg) and by 63.22% in the penumbra (0.064 ± 0.01 µmol/mg) compared with the control group. Moreover, administering the tolerant secretome led to a 34.63% reduction in the core and 65.03% reduction in the penumbra compared with administering the non-tolerant secretome (Fig. 7A). In addition, we observed a significant change in the glutamate blood levels in obese rats administered the tolerant secretome. In this particular group, glutamate increased by 224.70% (150.40 ± 5.31 µmol/l of blood) compared with the control group (46.32 ± 6.24 µmol/l of blood) and by 163.72% compared with the non-tolerant secretome group (57.03 ± 2.78 µmol/l of blood). The control group did not show any significant difference compared with the non-tolerant group (Fig. 7B).

#### The antioxidant effects of the blood cell–derived secretome in stroke-induced obese rats

**Fig. 8:**
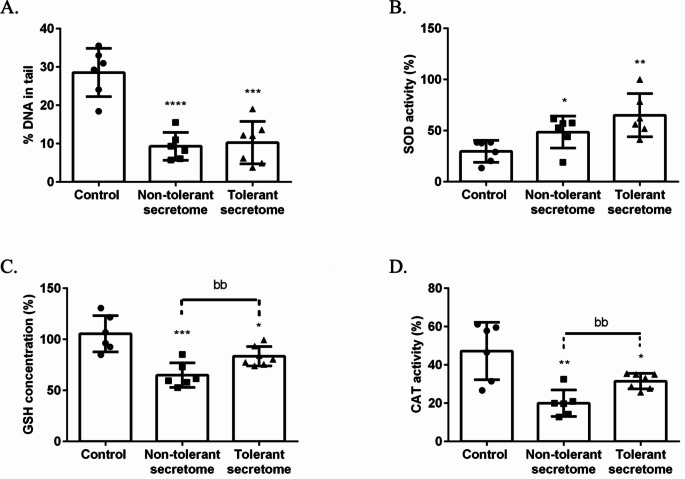
The histograms show quantification of (**A**) single-strand DNA breaks in lymphocytes and (**B**) SOD, (**C**) GSH, and (**D**) CAT activities in the blood of obese rats. The data are presented as the mean ± SD. *p < 0.05, *p < 0.01, ***p < 0.001 and ****p < 0.0001 versus the control group; ^bb^*p* < 0.01 versus the non-tolerant secretome group

We observed DNA damage in the control group (*n* = 6), denoted by 28.57% ± 2.57% DNA in tail. In contrast, the non-tolerant secretome (*n* = 6) and tolerant secretome (*n* = 7) groups (9.31% ± 1.49%; and 10.26% ± 2.09%, respectively) exhibited a significant decrease in DNA damage by about 67.41% and 64.09%, respectively (Fig. 8A). Furthermore, administration of tolerant and non-tolerant secretomes led to a significant increase in SOD activity (119.22% and 63.92%, respectively) compared with the control group (Fig. 8B). Additionally, compared with the control group (47.24% ± 6.14% and 105.5% ± 7.23%, respectively), the CAT and GSH activities were significantly decreased in the non-tolerant secretome group (reduced to 20.04% ± 1.53% and 65.00% ± 4.86%, respectively) and the tolerant secretome group (reduced to 31.52% ± 1.53% and 83.52% ±3.62%, respectively). However, there was a significant increase in the CAT and GSH activities in the tolerant secretome group compared with the non-tolerant secretome group (*p* < 0.0034 and *p* < 0.0098, respectively) (Fig. 8C, D).

## Discussion

Stroke is a highly complex and heterogeneous disorder caused by, or associated with, cardiovascular risk factors and comorbidities such as hypertension, diabetes, obesity, and non-modifiable risk factors such as age and the female sex (Tsao et al. 2023). Several preclinical studies on ischaemic stroke have shown that RIC is an effective endogenous neuroprotective phenomenon (Zhao et al. 2006; Xing et al. 2008; Yuan et al. 2011; Hu et al. 2012; Bonova and Gottlieb 2015; Jachova et al. 2019, 2020). However, comorbidities associated with stroke have been found to impair the response of ischaemic organs to RIC (Kersten et al. 2000; Bartling et al. 2003; Bouhidel et al. 2008; Kupai et al. 2009). In our previous study, we revealed that obese animals respond more severely to IR-induced brain injury compared with their lean healthy counterparts. Moreover, we discovered that the standard RIPostC protocol – that is, three cycles of 5 min of ischaemia with 5 min of reperfusion, which has been well documented to improve neurological outcomes after stroke in healthy cohorts – is ineffective in the ZDF rat model (Kotorová et al. 2024). In that study, we selected Zucker lean rats based on their phenotype at 12 weeks of age with no consideration of the genotype. We excluded Zucker lean animals from the current study because our main objective was to expand on the previously published results for obese rats. Given that the impact of RIC may vary by sex and age (Baig et al. 2021; Torres-Querol et al. 2021), we continued to use 12-week-old male rats to investigate obesity-related changes in RIC efficacy in the current experiment.

A single round of RIPostC, which is considered the gold standard of RIC-induced protection, may be insufficient to exert neuroprotective effects in patients with concomitant comorbidities. Therefore, we aimed to determine whether a second round of RIPostC after focal ischaemia could improve the neurological outcomes in obese animals that do not respond to the standard RIC stimulus. The preclinical study conducted by Ren et al. (Ren et al. 2015) supports the fact that repeated RIC may be a novel strategy to minimise the clinical impact of stroke. This view has also been supported by evidence from clinical trials in humans (Meng et al. 2012, 2015). Although a second round of RIPostC reduced the consequences of neurodegeneration in the penumbra caused by IR injury, our findings indicated that this approach did not significantly alter the infarct volume. Furthermore, there were no significant changes in glutamate blood and brain tissue levels. These findings are not entirely in line with the previously mentioned preclinical and clinical studies. However, it is important to mention that those studies employed heterogeneous groups of animals or humans of various ages, with the majority of the participants being healthy. In contrast, we showed that two rounds of RIPostC significantly decreased systemic oxidative stress by reducing oxidative DNA damage in circulating lymphocytes of obese animals induced by MCAO. Further analysis revealed enhanced SOD and GSH activities, but decreased CAT activity. Compared with administering a single round of RIPostC, our findings indicate that repeated occlusion and reperfusion cycles on the hind limb positively affects antioxidant defence system in obese animals with stroke.

We also evaluated the potential neuroprotective effects of the RIC-stimulated blood cell–derived secretome for obese rats with ischaemic stroke. The secretome represents a set of bioactive molecules released into the extracellular environment, which may play a significant role in numerous cellular processes, such as inflammatory modulation, tissue repair, and angiogenesis (Bruno et al. 2015; Gnecchi et al. 2016; Katagiri et al. 2017; Maacha et al. 2020). These molecules may be secreted by different cell types. Our group previously confirmed the neuroprotective effects of RIC-stimulated blood cells in an animal model of cerebral ischaemia (Bonova and Gottlieb 2015). We provided evidence that the neuroprotection could be transferred via the blood cell–derived secretome from rat to rat (Bonova et al. 2016, 2020). Based on previous findings from our laboratory showing that young lean rats release bioactive compounds in response to RIC treatment (Bonova et al. 2020, 2022), we hypothesised that these factors are transferred via the secretome to obese animals with stroke, potentially reducing stroke outcomes. Therefore, we followed the established protocol and used young, lean rats to produce the tolerant secretome, which we subsequently administered intravenously to ZDF rats.

We recently showed that the tolerant secretome reduced the infarct volume when applied before or after MCA occlusion in young healthy rats (Bonova et al. 2020). Other preclinical studies have explored the effect of extracellular vesicles of mesenchymal stromal cells, which are biologically active components found within secretomes, in animal models of ischaemic stroke. The administration of these vesicles improved functional recovery and reduced neurological impairment and the brain infarct volume (Giovannelli et al. 2023). However, the neuroprotective benefits of secretomes and their extracellular vesicles have been extensively studied using healthy rodents and there is a lack of research evidence supporting the use of these bioactive substances as a therapy of stroke in the context of obesity. We have shown for the first time that the tolerant secretome, when administered intravenously after cerebral ischaemia, reduced the infarct volume in obese rats by around 30% compared with the control group, and by around 25% compared with the group that received non-tolerant secretome. This intervention also improved the functional outcomes following stroke. In addition to the reduction in the infarct volume, the tolerant secretome attenuated neurodegeneration compared with the control and non-tolerant secretome groups, leading to a significant histopathological improvement in ischaemia-damaged neurons of the striatum and cortex within the penumbra.

It is well known that elevated glutamate brain levels contribute significantly to excitotoxicity, affecting neuronal survival in different brain regions (Bonova et al. 2013). Previous studies have shown that both pre- and post-treatment with RIC led to an increased release of extracellular glutamate from the ischaemic brain into the peripheral blood circulation (Bonova and Gottlieb 2015; Jachova et al. 2019, 2020; Končekova et al. 2023). Despite the application of an additional round of RIC, it seems that the accelerated brain-to-blood glutamate efflux is abolished in the presence of obesity. A decrease in some specific glutamate transporters in obese animals might contribute to the inability of RIC to enhance glutamate efflux from the brain to the blood (Liu and Zheng 2019). However, this hypothesis needs to be tested with additional experiments. According to a previous study (Bonova et al. 2020), bioactive substances released by RIC-stimulated blood cells have the ability to initiate protective mechanisms against toxic glutamate conditions in sensitive neuronal cultures in vitro. We observed a beneficial effect of the tolerant secretome on brain excitotoxicity induced by glutamate in vivo. Treatment with the tolerant secretome significantly reduced glutamate levels in the penumbra of ischaemic brain, along with a simultaneous increase in the blood glutamate concentration of obese rats.

Furthermore, we demonstrated that after administering the tolerant secretome to obese rats, there was reduced systemic oxidative stress after ischaemia, denoted by a reduction of DNA damage in peripheral blood lymphocyte. Surprisingly, we noted a similar effect in the non-tolerant secretome group. Based on our previous findings, the non-tolerant secretome has very modest the neuroprotective potential; however, on a much lower scale compared to those derived from RIC-treated individuals (Bonova et al. 2022). Although administration of the tolerant and non-tolerant secretomes led to a significant increase in SOD activity, the GSH and CAT activities decreased compared with the control group. However, we observed a significant increase in the activity of these enzymes after administration of the tolerant secretome relative to the non-tolerant secretome. We suggest that these enzymes are not the major determinants of RIC-stimulated secretome-induced neuroprotection. Nevertheless, antioxidant defence provided by the tolerant secretome, demonstrated by the suppression of DNA damage and the elevation of SOD activity in the blood, could contribute to the development of ischaemic tolerance in the brain of obese rats.

## Conclusion

In conclusion, the administration of the tolerant secretome showed neuroprotective benefits in obese animals with stroke, with a reduction in the infarct volume and neurodegeneration in the penumbra of ischaemic brain. Furthermore, the treatment enhanced brain-to-blood glutamate efflux and exerted antioxidant effects. Overall, these findings suggest that utilising the blood cell–derived secretome could represent an innovative approach to improve stroke outcomes, especially for patients with specific comorbidities, such as obesity. However, additional neuroprotective mechanisms that support our results and demonstrate the efficacy of the tolerant secretome in obese animals should be evaluated.

## Data Availability

No datasets were generated or analysed during the current study.
